# The electrical signature of mafic explosive eruptions at Stromboli volcano, Italy

**DOI:** 10.1038/s41598-022-12906-x

**Published:** 2022-05-31

**Authors:** Caron E. J. Vossen, Corrado Cimarelli, Alec J. Bennett, Markus Schmid, Ulrich Kueppers, Tullio Ricci, Jacopo Taddeucci

**Affiliations:** 1 Department of Earth and Environmental Sciences, Ludwig-Maximilians-Universität München, Theresienstraße 41, 80333 Munich, Germany; 2 Bristol Industrial and Research Associates Ltd (Biral), Unit 8 Harbour Road Trading Estate, Portishead, Bristol, BS20 7BL United Kingdom; 3 Department of Electronic and Electrical Engineering, University of Bath, Claverton Down, Bath, BA2 7AY United Kingdom; 4 Istituto Nazionale di Geofisica e Vulcanologia, Sezione di Roma 1, Rome, Italy

**Keywords:** Atmospheric dynamics, Geophysics, Volcanology

## Abstract

Volcanic lightning is commonly observed in explosive volcanic eruptions of Volcanic Explosivity Index (VEI) > 2 and can be detected remotely providing real-time volcano monitoring information. However, little is known about the electrical activity accompanying the lower-magnitude spectrum of explosive eruptions, often involving mafic magmas. We narrow this gap in knowledge by presenting the electrical signature of the explosive activity (VEI ≤ 1) of Stromboli volcano (Italy) recorded by an electrostatic thunderstorm detector. The persistent eruptive activity of mild Strombolian explosions is occasionally interrupted by larger-scale major explosions and paroxysmal events.

Here, we present electrical observations of three major explosions and unprecedented measurements of the 3 July 2019 paroxysm. The electrical signals of the major explosions show apparent similarities, with movements of charge and tens of electrical discharges, arising the question of whether these observations could be used to supplement the classification scheme of explosions on Stromboli. The electrical signals from the 3 July 2019 paroxysm exceed those from the major explosions in amplitude, discharge rate and complexity, showing characteristic variations during different phases of the eruption.

These results show that also impulsive lower-magnitude explosions generate detectable electrical activity, which holds promise for monitoring low VEI activity at mafic volcanoes.

## Introduction

### Electrical activity and lightning in volcanic plumes

Explosive volcanic eruptions generate changes in the electric field and volcanic lightning^1^. An important component of plume electrification is the presence of silicate particles, which are considered the main carrier of electrical charge in volcanic jets and plumes. Upon explosion, the fragmentation of magma into pyroclasts^2,3^ (fragments of silicate melts) and their subsequent collisions^4–6^ generate high electrical charge in the expanding volcanic jets. Under specific conditions, also ice nucleation/riming^7,8^, interaction with (sea)water^9–11^ and, to a lesser extent, natural radioactivity^12,13^ may contribute to plume electrification. Besides these external effects, the distribution of charges in the evolving plume creates the conditions for electrical discharges to occur^1^.

Volcanic plume electrification and lightning is commonly observed at volcanoes characterized by a Volcanic Explosivity Index (VEI) > 2, therefore being generally associated with intermediate to high-silica magma compositions^1,14^. The occurrence of volcanic lightning during explosive eruptions of basaltic composition was reported for a wide range of plume heights (1–21 km), although considerably fewer reports were found for eruptions of VEI ≤ 1^15^, such as Strombolian explosions^14^. Albeit experiencing brittle fragmentation^16^, the reduced ability of such magmas to produce lightning activity is generally attributed to the low viscosity and high temperature of low-silica magmas which promotes outgassing and gas-magma decoupling, often resulting in effusive eruptions or mild explosions^17^. However, specific processes, such as molten fuel–coolant interaction (MFCI)^10^, the combination of strong magma foaming and geometrical obstructions^18^, the obstruction of the conduit through talus accumulation^19^ or the presence of a magma plug, can result in enhanced explosivity and consequently generate more volcanic lightning.

High-temperature experiments have investigated the production of electrical signals of basaltic magma upon fragmentation^10,20^. The experiments indicate that formation of new surface area and subsequent particle cloud expansion generate charge separation, which can be detected on a short timescale as an electrostatic field gradient. Electrostatic field gradients comparable to those measured in the experiments were detected during mild Strombolian explosions^20,21^. Additionally, an electric potential gradient accompanied by electrical discharges was recorded during an ash-rich major explosion on 7 September 2008^21^.

To further increase our knowledge of electrical activity accompanying basaltic eruptions, we carried out long-term electrostatic field measurements on Stromboli volcano, Italy.

### Study area

**Figure 1:**
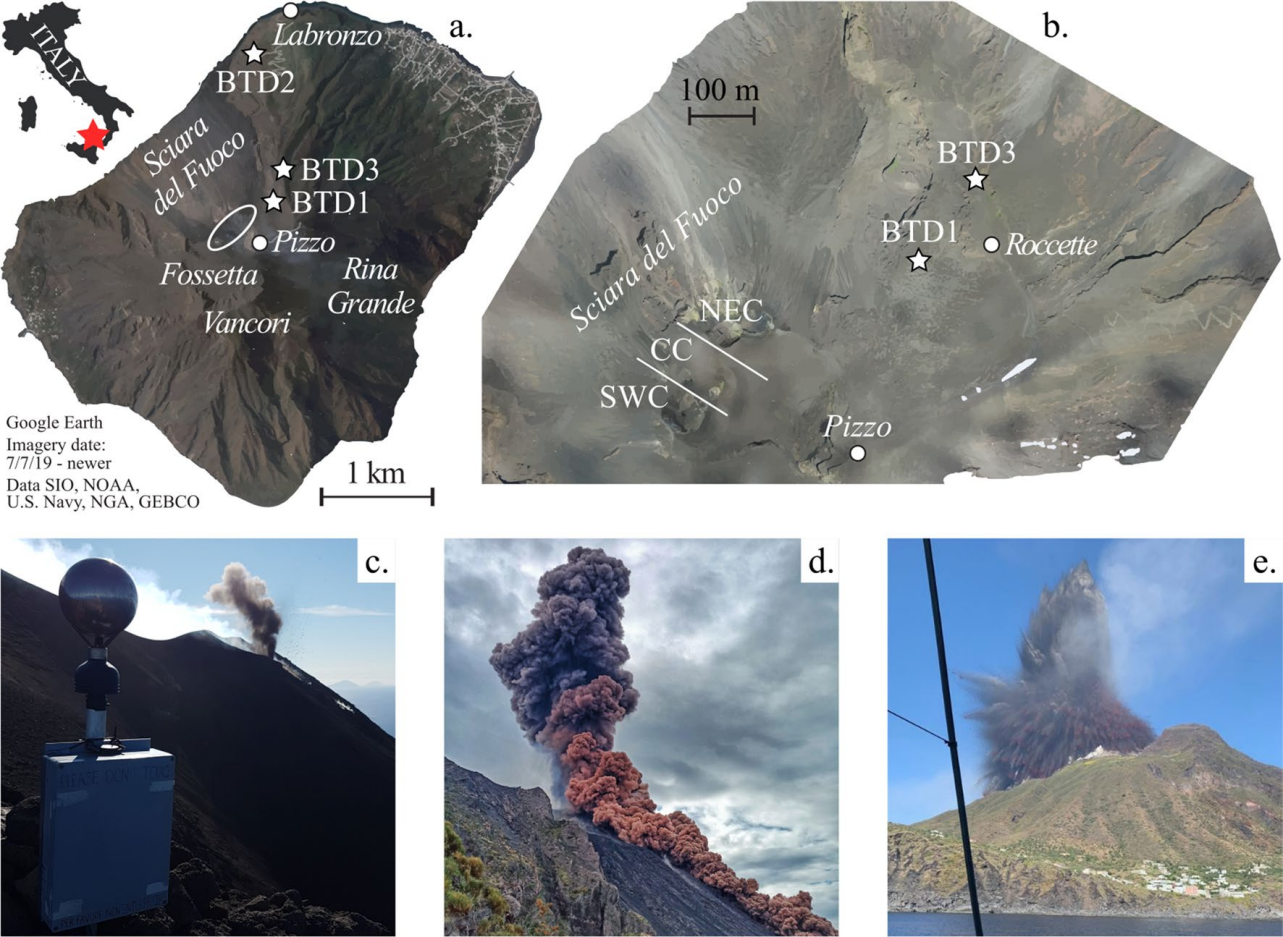
(**a**) Map of Italy (Inset), showing the location of Stromboli island (red star), together with a satellite image from Google Earth (Imagery date: 7/7/2019—newer. Data SIO, NOAA, U.S. Navy, NGA, GEBCO. http://www.earth.google.com [2 June 2021]). The location of the crater terrace (780 m a.s.l., encircled in white), the three succeeding locations of the sensor (BTD1, BTD2 and BTD3, star symbols), and important localities (Pizzo (918 m a.s.l.), Vancori (924 m a.s.l.), Punta Labronzo, Sciara del Fuoco, Rina Grande and Fossetta) are indicated. (**b**) Drone image of the crater terrace, showing the Northeast Crater (NEC), Central Crater (CC) and Southwest Crater (SWC) zones, and the locations of BTD1 and BTD3 close to Roccette. The drone flight was carried out in June 2019^25^. (**c**) BTD3 installed close to Roccette on 9 October 2020. A Strombolian explosion (type 2b^39^) from the NEC zone is visible in the background. (**d**) Photograph of the 16 November 2020 major explosion. A large part of the pyroclastic density current was the result of the collapse of the crater rim, evident from the reddish colour. Courtesy of Adriano Di Pietro. (**e**) Photograph of the 3 July 2019 paroxysm taken from a sailing boat. Courtesy of Francesco Rinauro.

Stromboli volcano is well known for its persistent mild explosive activity since historic times^22^, for which it is also referred to as “the lighthouse of the Mediterranean” (Fig. 1a). Although the number of active vents changes frequently, the crater terrace of Stromboli consists of three main crater zones, the Northeast (NE), Central (C) and Southwest (SW) crater zones (Fig. 1a,b)^23^. Due to its constant explosive conditions, Stromboli is closely monitored by permanent geophysical networks of the Laboratorio di Geofisica Sperimentale (LGS) of the University of Florence and the Istituto Nazionale di Geofisica e Vulcanologia (INGV) including seismometers, an infrasonic array, tiltmeters, dilatometers, ground-based interferometric synthetic aperture radars, and visible and thermal cameras^24^.

### Volcanic activity at Stromboli

Stromboli is characterized by continuous active degassing (puffing) alternated with mild explosions (VEI ranging from − 6 to − 3^15,26^) that episodically eject variable proportions of volcanic ash and incandescent ballistic particles to heights of tens to hundreds of metres every few minutes^27,28^. This Strombolian activity, also referred to as “normal activity”, is fed by a degassed, highly crystallised, high porphyritic (HP) magma of basaltic composition stored in the shallow (< 2 km depth) plumbing system^29–32^. The normal activity is occasionally interrupted by lavas flowing down the Sciara del Fuoco as well as major explosions (VEI ranging from − 3 to 0^26^) and paroxysmal events (VEI = 0–1^15,26^).

Major explosions occur on average twice per year^23,33^ (Fig. 1c). During these eruptions, multiple vents of one or more craters simultaneously eject decimetre-sized ballistic blocks and bombs within a distance of hundreds of metres from the crater terrace^31,34^. The plumes are short-lived and can reach up to one kilometre height above the crater rim^35^. The subsequent fallout of ash and light-lapilli is typically restricted to the slopes of the volcano^31,34^. The deposits commonly consist only of HP magma.

In the last 140 years, a paroxysmal event occurred on average every 3.85 years^33^ (Fig. 1d). They are similar to major explosions, but larger in magnitude, more violent and often accompanied by pyroclastic flows^23,35^. Paroxysms occur when deep (7–10 km depth) volatile-rich, crystal-poor, low porphyritic (LP) magma of basaltic composition rapidly ascends to the surface, increasing the pressure either due to bubble growth upon decompression or the accompanying rise of a gas slug^29–32^. Deposits from the 1930, 2003 and 2007 paroxysms consist of golden-coloured pumices, products of the LP magma, mixed with black scoriae, products of the HP magma, indicating that the two magmas mingled shortly before and during the eruption^29–32^. Typically all active vents are involved from which metre-sized ballistic scoria bombs and blocks are ejected up to several kilometres from the crater terrace^23,31,36^. The convective plume rises to several (> 3–4) kilometres in height, followed by tephra fallout beyond the coastline^23,31,36,37^.

Here, we present the electrical signals recorded during three selected major explosions within 1.5 years, and the 3 July 2019 paroxysmal event. Although volcanic lightning was reported during the 15 March 2007 paroxysm^38^, it has never been measured during one of the paroxysmal events at Stromboli until now. Note that we use the terms volcanic lightning and electrical discharge interchangeably to describe the detected signals.

**Table 1:** Eruption parameters and electrical measurements for the 25 June 2019, 19 July 2020 and 16 November 2020 major explosions and the 3 July 2019 paroxysm.

	Major explosion 25 June 2019	Major explosion 19 July 2020	Major explosion 16 Nov 2020	Paroxysm 3 July 2019
Onset time (UTC)	23:03:08^35,41^	03:00:42 03:01:11 03:01:28^35,42^	09:17:45^35,43^	14:45:43^35^ 14:45:53^45^
Active crater zone	CC^35,41^	CC, SWC^35,42^	NEC, CC, SWC^35^	SWC, NEC^35,45^
Acoustic pressure (Pa)	170^44^	> 1500^46^	562^47^	N.A
Maximum particle ejection velocity (m/s)	54.41^35^	78.22^35^	54.51^35^	101.92^35^
Plume height a.c.r. (m)	~ 500^35^	> 750^35^	1000^43^	8400^37^
Explosion duration (s)	8^35^	58^35^	54^35^	160^35^
Onset of electrical activity (UTC)	23:03:08.08	03:00:43.8	09:17:44.87	14:45:43.63
Time until last discharge (s)	7.32	68.2	13.13	> 44.74
Number of discharges	37	80	49	> 321
Maximum discharge rate (discharges/5 s)	24	20	33	49
Distance between active crater(s)—sensor (m)	403	1556–1649	425–616	292
Peak value of charge movement at sensor (V)	0.0012	0.00065	0.033	0.023
Peak value of charge movement at active crater (V)	7.85 × 10^4^	2.45 × 10^6^–2.91 × 10^6^	2.53 × 10^6^–7.71 × 10^6^	5.73 × 10^5^
Charge moment (mC m)	0.2–2.0	20–150	100–200	20–1000

## Results

More than 40 Strombolian explosions of varying types (1, 2a and 2b^39^) were recorded on 11 and 12 June 2019 by one of the Biral Thunderstorm Detectors (BTD1). An example of each type is shown in Supplementary Figure S1^40^. During most of these explosions, the primary antenna recorded slow (~1–3 s) electrostatic variations. In an aerosol-rich environment, such as a volcanic eruption, the majority of space charge will get attached to the particles, with a smaller proportion of ions bound to the volcanic gases^13^. The movement of charge as a result of the ejection of charged volcanic products generates these slow-varying electrostatic signals. Some of the ash-rich explosions additionally showed transient-like signals, the steep onset and decrease indicating a short-lived, sharp change in the electric field, which correspond to small discharges following the explosions. Although the results indicate that these explosions produce faint detectable electrical signals, the wide variety of explosions (regarding the size of pyroclasts, ejection velocity and bulk volume) impedes our understanding of the origin of these signals and requires a more thorough analysis. For this reason, the rest of this study will only focus on the electrical activity produced by the larger explosions at Stromboli.

The sensor recorded a major explosion at each location: the 25 June 2019 major explosion (BTD1), the 19 July 2020 major explosion (BTD2) and the 16 November 2020 major explosion (BTD3)^40^. Additionally, BTD1 recorded the 3 July 2019 paroxysm^40^ (Fig. 1d). Detailed information on each explosion was gathered, inter alia, from the weekly monitoring bulletins provided by INGV on www.ct.ingv.it and LGS on http://lgs.geo.unifi.it/, and recently collected on the site https://cme.ingv.it/bollettini-e-comunicati (Table 1).

### The 25 June 2019 major explosion

**Figure 2:**
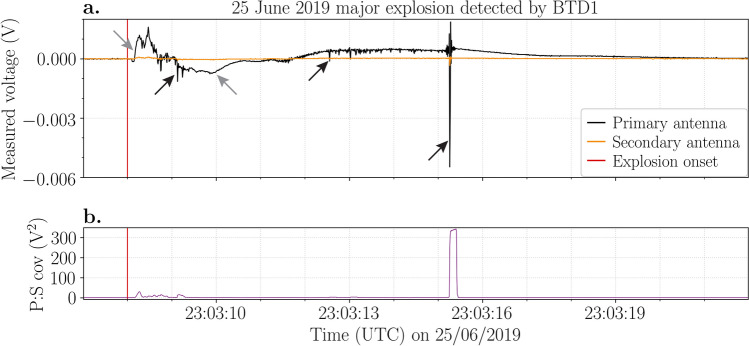
Electrical data of the 25 June 2019 major explosion recorded by BTD1. (**a**) Voltage (V) measured by the primary (black line) and secondary (orange line) antennas. Examples of movement of charge and electrical discharges are indicated by the grey and black arrows, respectively. (**b**) The covariance (V^2^) between the primary and secondary signals. The red vertical line indicates the explosion onset.

A major explosion occurred on 25 June 2019 at 23:03:08 UTC^35^. At 23:03:08.08 UTC (Table 1), BTD1 recorded a positive variation in charge, which changed polarity one second later (Fig. 2a). This was followed by small electrical discharges shortly after (Fig. 2a), which is evident by the positive covariance (Fig. 2b). Approximately 7.3 s after the explosion onset, the last discharge was detected, which had a measured voltage an order of magnitude higher than the preceding ones. The electrical signal returned to pre-explosion background levels in about 48 s. The entire electrical perturbation caused by the eruption lasted 54 s.

The strength of the electrical discharges is indicated by their charge moment magnitude, which is proportional to the total charge neutralised by the discharge. Estimates of this value can be found from the current induced on BTD1, which when integrated with time is proportional to the total charge transfer. Assuming the discharges occurred at low-level above the active crater, the charge moment magnitudes from discharges during this major explosion ranged from 0.2 to 2.0 millicoulomb metre (mC m) (Table 1).

### The 19 July 2020 major explosion

**Figure 3:**
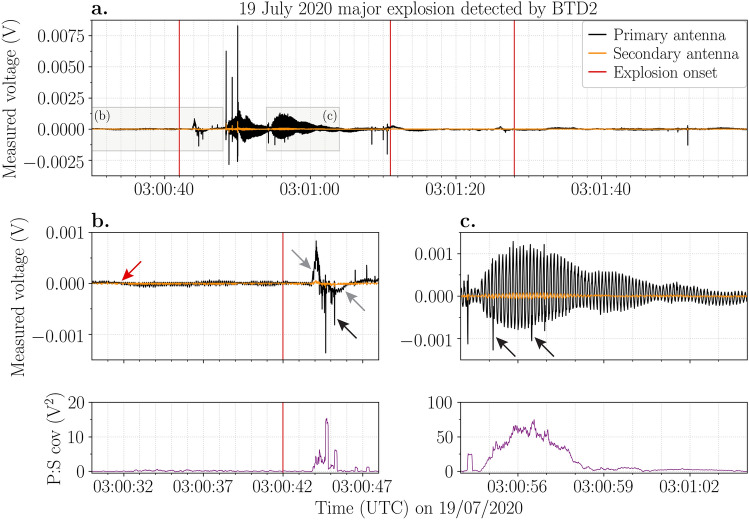
Voltage (V) measured by the primary (black line) and secondary (orange line) antennas of BTD2 during the 19 July 2020 major explosion. (**a**) Electrical signal detected during three separate explosions (red vertical lines) at 03:00:42 UTC, 03:01:11 UTC and 03:01:28 UTC. (**b**) An oscillating electrostatic signal starts approximately 10 s before the explosion onset (red arrow). Shortly after the onset, movement of charge (grey arrows) and electrical discharges (black arrows) were detected. (**c**) The amplitude of the oscillation is correlated to the magnitude of the charge variation. Electrical discharges are superimposed on the oscillations (black arrows). The corresponding covariance (V^2^) between the primary and secondary signals is shown below for panels (**b**) and (**c**).

Even though BTD2 was installed at a much greater distance from the crater terrace, it successfully detected the electrical activity generated by the 19 July 2020 major explosion. The explosion onset was estimated at 03:00:42 UTC^35^. A positive charge variation was detected at 03:00:43.8 UTC (Table 1), promptly followed by small electrical discharges (Fig. 3a,b). Several discharges with higher measured voltages were detected approximately 5 s later. At 03:01:11 UTC a second explosion occurred, accompanied by a new movement of charge, although of slightly lower magnitude than the one associated with the first explosion. A third explosion occurred at 03:01:28 UTC, but no clear electrical signal was detected at this point. In total, these three explosions lasted 58 s^35^. However, INGV reported that this phase was followed by several smaller explosions^42^, which likely explains the three discharges detected at around 03:01:52 UTC. Afterwards, the electrical signal continued to vary for several minutes. The charge moment magnitudes from the discharges generated by this major explosion were estimated to range from 20 to 150 mC m (Table 1), assuming they occurred at low-level above the crater rim.

In addition to detecting electrical discharges and movement of charge, an oscillating signal with a frequency of 8–9 Hz was observed in both the primary and secondary antenna time-series for the whole eruption (Fig. 3). This oscillating signal started at 03:00:31.7 UTC (Fig. 3b), approximately 10 s before the explosion onset, and shows time correlation with the first peak in seismicity recorded by the INGV^35^ and LGS seismic stations. The signal amplitude correlates positively with the magnitude of charge variation (Fig. 3c), while electrical discharges are superimposed.

### The 16 November 2020 major explosion

**Figure 4:**
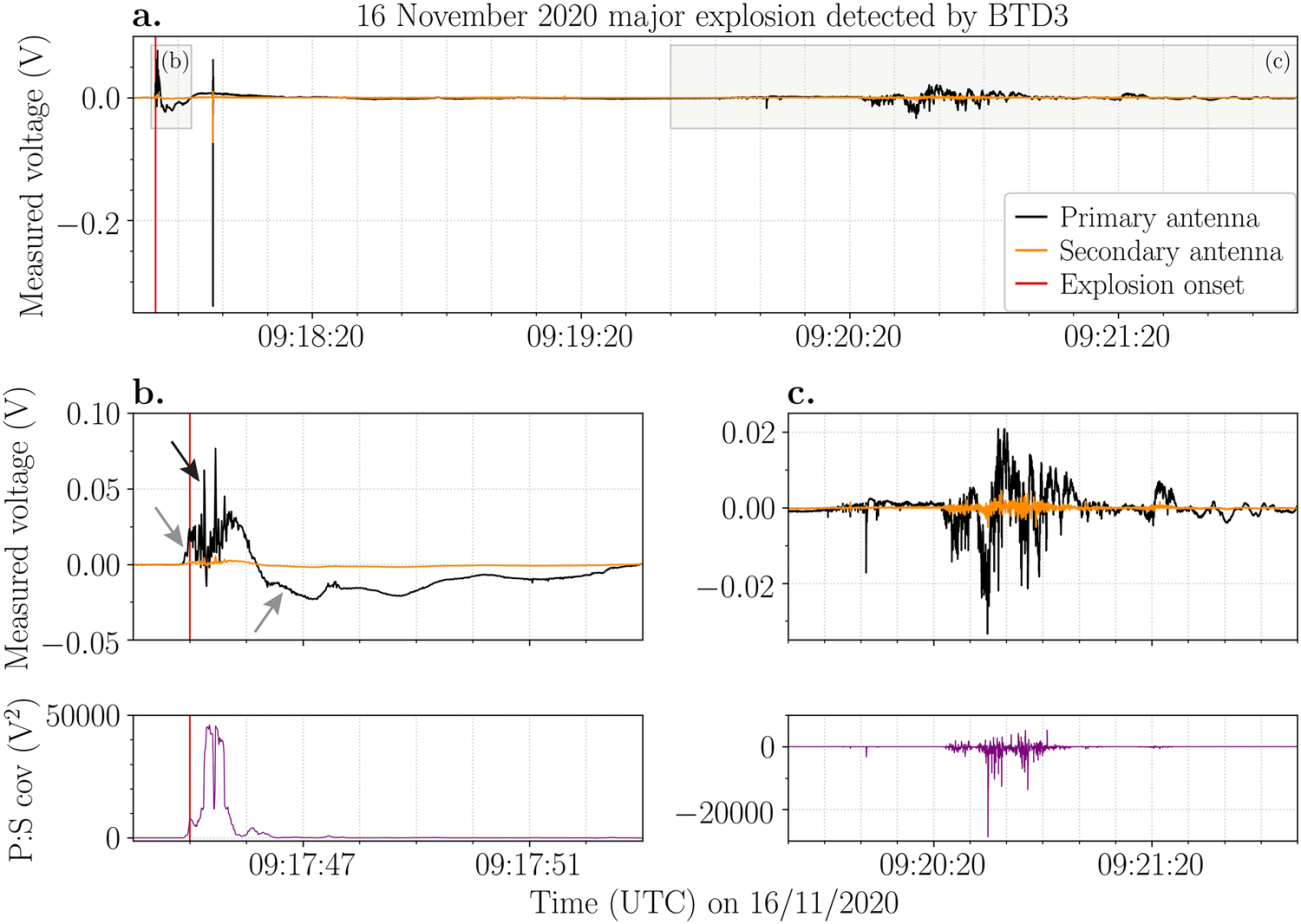
(**a**) Voltage (V) measured by the primary (black line) and secondary (orange line) antennas of BTD3 during the 16 November 2020 major explosion. The red vertical line indicates the explosion onset. (**b**) Movement of charge (grey arrows) and electrical discharges (black arrow) were detected almost immediately at the start of the explosion. The corresponding covariance (V^2^) between the primary and secondary signals is shown below. (**c**) Electrical signal likely caused by ash impact transients as confirmed by the corresponding negative covariance (below).

The 16 November 2020 major explosion produced an electrical signal similar to the 25 June 2019 explosion (Fig. 4a). Movement of charge was detected at 09:17:44.87 UTC (Fig. 4b), which corresponds well to the estimated explosion onset at 09:17:45 UTC^43^ (Table 1). Electrical discharges started to occur almost simultaneously but decreased in frequency and magnitude 2 s later (Fig. 4b). Assuming the discharges occurred at low-level above the active crater, the charge moment magnitudes from discharges during this major explosion ranged from 100 to 200 mC m (Table 1). Approximately 13 s after the eruption onset, a single electrical discharge occurred, with a measured voltage about one order of magnitude higher than the preceding discharges (Fig. 4a).

At 09:20:00 UTC an electrical signal with dominantly negative covariance values was recorded (Fig. 4c), indicating impact transients. INGV published images from monitoring cameras showing a pyroclastic density current (PDC) flowing down the Sciara del Fuoco seconds after the onset^43^. Subsequently, both the ash plume caused by the explosion and the ash cloud produced by the PDC seem to move in a west-southwest direction. This is confirmed by weather data obtained from station LICT at Trapani Airport (Sicily, Italy) provided by the University of Wyoming, Department of Atmospheric Science (http://weather.uwyo.edu/). At 12:00 UTC the wind direction varied between 220° and 270° at heights ranging from sea level to the maximum height of the ash plume (1000 m a.c.r., Table 1). Hence, it is probable that ash fell at BTD3, producing a signal of opposite polarity at the two antennas.

Note that the peak change in charge and the measured voltages of the electrical discharges produced by the 16 November 2020 major explosion are in general 1–2 orders of magnitude higher than those produced by the 25 June 2019 major explosion, even though BTD3 was located further away from the active craters with respect to BTD1.

### The 3 July 2019 paroxysm

A paroxysmal event occurred on 3 July 2019 (Fig. 1d). Although a posteriori analysis indicated the occurrence of anomalous seismic signals and heightened CO_2_ emissions months before the paroxysm^48,49^, no clear precursory signals were detected by INGV and LGS at the time of the eruption. Both institutes classified the volcanic activity in the week preceding the paroxysm as “moderate”.

Preceded by lava extruding from all vents, the paroxysm started with a localised explosion at 14:45:43 UTC at the SW crater zone and was followed by a second explosion at 14:45:53 UTC at the NE crater zone^45^. Decimetre-sized ballistics started falling close to Roccette 7 s after the start of the explosion, lasting for at least 7 s^37^. This was based on images taken by the Skyline webcam located at Roccette, approximately 100 m further away from the crater terrace than BTD1. Pictures of the eruption column initially suggested a plume height of 5 km above the summit of the volcano, but it was later estimated that the umbrella cloud reached a height of 9180 m a.s.l.^37^. The latter agrees with the observation of the Volcanic Ash Advisory Center (VAAC) Toulouse, which reported the presence of ash at flight level FL300. A flash of volcanic lightning is visible in the top part of the convective plume in a video taken offshore by a witness. Portions of the eruption column and the crater terrace outer rim collapsed, forming at least two PDCs down the Sciara del Fuoco, which reached the sea at 14:46:29 UTC and 14:46:39 UTC, respectively, based on the LGS monitoring camera at Punta Labronzo^45^. They continued to travel for approximately 1 km beyond the coastline.

**Figure 5:**
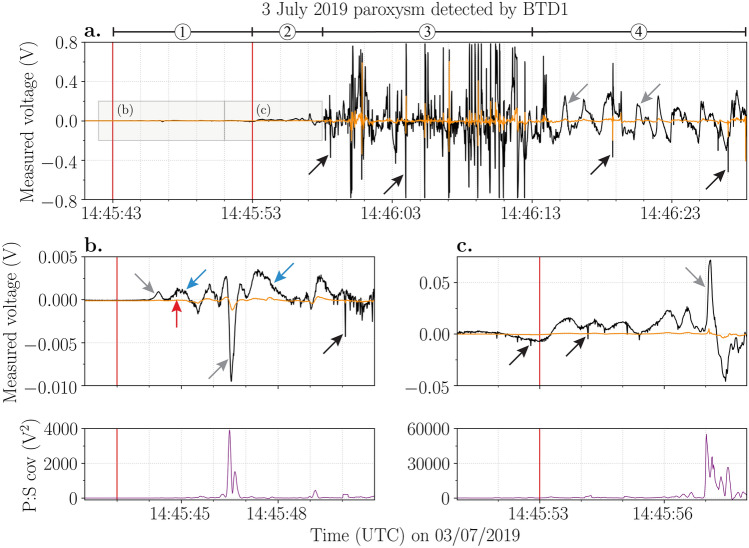
Voltage (V) measured by the primary (black line) and secondary (orange line) antennas of BTD1 during the 3 July 2019 paroxysm. The red vertical lines pinpoint two separate explosions at 14:45:43 and 14:45:53 UTC, respectively. Examples of movement of charge and electrical discharges are indicated by the grey and black arrows, respectively. (**a**) The entire electrical signal of the paroxysm until the power cut. Four different phases (1–4) of electrical activity can be distinguished. (**b**) The electrical signals recorded during the first explosion with their corresponding covariance (V^2^) below. The red arrow marks the first electrical discharge and the blue arrows mark small irregular variations in the electrical signal likely caused by pyroclasts moving towards BTD1. (**c**) The electrical signals recorded during the second explosion with their corresponding covariance (V^2^) below. Note the different scale on the y-axis of each panel.

The BTD1 remained visibly unscathed during the eruption, providing us with unprecedented electrical observations of the paroxysm. The paroxysm lasted 160 s^35^ (Table 1), of which BTD1 recorded 45.35 s. The electrical activity generated by this event was much more intense compared to the previously discussed major explosions. Four phases of electrical activity can be distinguished (Fig. 5):

1. A slow varying electrostatic signal started at 14:45:43.63 UTC (Fig. 5b, Table 1), which corresponds to the moment incandescent lava fragments emerged from the SWC zone and expanded radially (Fig. 1d), based on the images of the INGV thermal cameras at Pizzo^49^. Within a second, small irregular electrical variations were detected, followed by the first electrical discharge at 14:45:44.85 UTC. Electrical discharges became more frequent after 14:45:50 UTC.
2. After the second explosion at 14:45:53 UTC, both the magnitude of charge being moved and of the electrical discharges increased by more than one order (Fig. 5c). The peak charge change detected directly after the first explosion was approximately 0.0009 V, which increased by more than 25 times (0.023 V) immediately after the second explosion. We need to consider, however, that the second explosion occurred at the NEC zone, 177 m closer to BTD1 than the first explosion from the SWC zone. The difference in distance accounts for an increase in magnitude of approximately four times, indicating that the change in magnitude is not solely due to the shorter distance. Note that during the two explosions, higher peak values were reached, but to compare it to the major explosions, we took the initial movement of charge caused by the explosion.
3. At 14:45:58 UTC, the electrical activity evolves into a high-intensity phase. Here, the measured voltages of the electrical discharges caused the primary antenna to saturate, meaning their measured voltage exceeded 0.78 V (Fig. 5a).
4. This is followed by an abrupt change in signal around 14:46:13 UTC, with mostly low voltage discharges (occasionally interrupted by a larger discharge) and a relatively slow varying, high magnitude movement of charge (Fig. 5a). This signal continued until the power was cut off approximately 15 s later.

The charge moment magnitudes from discharges during this paroxysm ranged from 20 to 1000 mC m (Table 1), although the vertical extent of the plume increases the uncertainty of discharge altitude, making these estimates more likely to be an underestimation. These discharges were therefore at least 500 times stronger than those recorded during the 25 June 2019 major explosion and over 10 times stronger than the 19 July and 16 November 2020 major explosions, but still weaker than lightning typical of meteorological thunderstorms.

## Discussion

To our knowledge, none of the global thunderstorm networks recorded any volcanic lightning during the three major explosions and paroxysmal event discussed here. On the contrary, our thunderstorm detector was able to detect many electrical discharges and strong movements of charge, regardless of its position on Stromboli. These results show that sufficient charging occurs during the fragmentation of hot basaltic magma to result in volcanic lightning, albeit with a substantially smaller charge moment magnitude than lightning typically observed during thunderstorms.

### Major explosions

The similarities between the electrical signals of the three major explosions are evident (Figs. 2, 3, 4), especially for the 25 June 2019 and 16 November 2020 events. The ejection of the gas-particle mixture and consequent expansion of the jet produced a strong movement of charge, which was promptly detected by the sensors at the start of each explosion. This shows that electrical signals could be valuable in pinpointing the start of the explosions. The accompanied plume electrification resulted in many discharges of low measured voltage almost immediately after the onset. Subsequently, the movement of charge reversed polarity due to the rapid movement of charged gases and particles during the initial burst. These signals are comparable to the signal detected during the 7 September 2008 major explosion^35^. This could indicate a self-similar dynamic of such explosions where the mechanism of fragmentation, particle ejection velocity and erupted mass are alike.

The single electrical discharge detected seconds after the start of the 25 June 2019 and 16 November 2020 major explosions likely occurred to neutralise most of the remaining charge and therefore had a higher measured voltage than the previous discharges. The 19 July 2020 major explosion produced more of these high voltage discharges. The first two main explosions of the 19 July 2020 major explosion generated a movement of charge that was detected by BTD2 (Fig. 3a). INGV reported that the second and third explosions were less intense than the first^42^. This observation fits with the electrical data. The magnitude of the charge change detected during the second explosion is lower than the first, while no electrical activity was detected at all during the third explosion (Fig. 3a). A possible explanation for the absence of electrical activity could be that the sensor was not able to detect the weak change in charge generated by the third explosion at such a great distance from the craters. Note, however, that a small movement of charge was detected at ~ 03:01:26 UTC (Fig. 3a). As images from monitoring cameras sometimes make it difficult to pick the exact explosion onset, it could be possible that this is the true onset of the third explosion.

Although the maximum particle ejection velocities of the 25 June 2019 and 16 November 2020 major explosions were almost equivalent, the explosion duration was longer and the plume height was twice as high for the latter (Table 1)^35^. This suggests a higher magnitude, also evident from the higher acoustic pressure, and a greater erupted mass for the 16 November 2020 explosion, which would result in stronger plume electrification. This scenario agrees with our results, as both the measured voltages of the electrical discharges and the corrected peak charge variation were 1–2 orders of magnitude higher during the 16 November 2020 explosion (Figs. 2, 3). Also the number of discharges, the estimated charge moment magnitude, the maximum discharge rate and the duration of electrical activity were higher (Table 1). In addition to its higher magnitude, the PDC that followed the 16 November 2020 explosion, which largely resulted from the collapse of the crater rim as is evident from its reddish colour (Fig. 1d), likely generated additional electrification, resulting in more electrical discharges.

The number of discharges, as well as the duration of electrical activity, were highest for the 19 July 2020 explosion. This is not surprising, as this explosion consisted of multiple ejection pulses over several minutes instead of a single explosion. Each pulse would generate renewed electrification by ejecting new material. Even though the maximum particle ejection velocity was highest for the 19 July 2020 explosion, the maximum discharge rate was lower than for the other two major explosions. These results suggest that the eruptive mass and the total duration of the explosion play a more important role in the generation of electrical activity than the maximum particle ejection velocity.

There is some debate about whether the 19 July 2020 explosion should be classified as a major explosion or a paroxysmal event. In one study, volcanological and geophysical monitoring data were combined with remote sensing techniques to classify explosions of variable intensity^35^. They classified the 19 July 2020 explosion as a paroxysmal event based on two parameters, the Very Long Period (VLP) size^48^ and the power of explosive activity (VD parameter)^35^. On the other hand, INGV and LGS both classified it as a major explosion, based on the magnitude of several geophysical parameters (seismicity and deformation) as well as the dispersion of pyroclasts restricted to the slopes of the volcano^42,46^. Moreover, the contained plume height would rather fit the size of a major explosion (Table 1). The electrical signal accompanying this event shows more similarities with the signals recorded during 25 June 2019 and 16 November 2020 major explosions and lacks the high-intensity phase of electrical discharges characterising the paroxysm (phase 3, Fig. 5). The maximum ejection velocity, the total explosion duration and the plume height were considerably lower than for the paroxysmal event (Table 1). As a consequence, the 19 July 2020 explosion likely had weaker plume electrification and smaller charge regions, resulting in less intense electrical activity. Therefore, based on electrical signals, the 19 July 2020 explosion would fit the rank of a major explosion. Additional electrical data of both major and paroxysmal explosions at Stromboli would supplement or even elaborate further the classification scheme of such events.

Of particular interest is the oscillating signal characterising the 19 July 2020 explosion (Fig. 3b). Oscillating signals can be caused by strong winds or ground movement. In the latter case, the antennas themselves are rapidly moving in the electric field (due to ground-shaking), thereby producing a displacement current in the antenna. The stronger the electric field, the higher the amplitude of the oscillations, as is evident from the electrical data (Fig. 3c). Seismic signals recorded by INGV^35^ and LGS show an increase in peak amplitude about 10–15 s before the main peak associated with the explosion. This coincides with the start of the oscillations in the data at around 03:00:31.7 UTC. Hence, we conclude that the seismic tremor associated with the 19 July 2020 major explosion was recorded in the electrostatic data as a result of the oscillating antennas in the presence of an increased electric field, creating an important link between these two geophysical parameters. As volcanic lightning can be recorded by seismometers in the form of spikes or simultaneous gain ranging^51^, we argue that ground-shaking accompanying explosive eruptions may be recorded by electrical sensors coupled to the ground.

### The 3 July 2019 paroxysm

Four different phases of electrical activity were distinguished within the 45 s of data recorded during the 3 July 2019 paroxysm (Fig. 5), which can be tentatively matched to different phases of the eruption:

1. The slowly varying electrostatic signals recorded following the first explosion were caused by the movement of charge due to the growing and rupturing of a metre-sized lava bubble (Fig. 5b), as shown by thermal images from the INGV monitoring cameras^49^. The small irregular electrostatic variations detected by the primary antenna one second later, were likely caused by weakly charged pyroclasts moving towards and landing in front of the sensor. Their weak charge was insufficient to induce a detectable opposite effect on the secondary antenna. The movement of charge reversing to a negative polarity in all probability corresponds to the radially expanding, pyroclast-rich eruption jet passing over the sensor and moving away from it.
2. After the second explosion, the magnitude of the charge change and the discharges increased by an order of magnitude (Fig. 5c). We showed that the shorter distance to the NEC zone only accounts for a small increase in the magnitude measured by the sensor. Instead, the additionally erupted material, which encountered an already ash-contaminated and charged atmosphere, enhanced the plume electrification and consequently generated discharges of higher magnitude^5,52^.
3. While the plume continued to rise and grow by convection, the turbulence caused charged clusters to form, further increasing the magnitude of the electric field^53^. As a consequence, electrical discharges became more frequent (reaching the maximum detected discharge rate) and produced higher measured voltages (Fig. 5a), saturating the primary antenna. Whilst these discharges were the strongest recorded during this campaign, their estimated charge moment magnitudes of approximately 1000 mC m (0.001 C km) were still weaker than that expected for thunderstorm lightning, which is typically 10–100 C km. It is however acknowledged that the taller plume during the paroxysm and temporary saturation of the BTD antennas increase uncertainty of these charge moment estimates.
4. The abrupt shift in the electrical signal from discharges of high voltages to low voltages at 14:46:13 UTC (Fig. 5a) seems to coincide with partial collapse of the plume and the subsequent PDCs flowing down the Sciara del Fuoco, based on visible images taken by the LGS monitoring camera at Labronzo locality^54^. The partial collapse likely resulted in charge removal from the volcanic plume, similar to the dissipating stage of a thunderstorm, where the downdraft cuts off the updraft of a thunderstorm, weakening it and ceasing meteorological lightning^55^. The turbulence of the PDCs generated new electrification. The movement of charge caused by the fast-moving PDCs close to the sensor produced the slow varying electrostatic signal of high magnitude. The charge clusters within the PDCs are probably smaller compared to those in the volcanic plume. As these regions are less extensive, electrical discharges of smaller magnitude are expected to occur, which is consistent with the last 15 s of the electrical data recorded by BTD1. It is uncertain whether the occasional discharge of higher measured voltage occurred within the volcanic plume or the PDCs.

The electrical activity detected during the 3 July 2019 paroxysm was much more vigorous, in both magnitude and discharge rate, and complex compared to the electrical signals detected during the major explosions. The eruption duration, plume height and maximum ejection velocity were significantly higher for the paroxysmal event (Table 1), indicating higher material ejection rates. This would have greatly attributed to the plume electrification, resulting in more electrical discharges with a larger magnitude. Despite this, the first electrical discharge occurred at a later time with respect to the explosion onset when compared to the three major explosions. Thermal images from monitoring cameras^50,54^ showed lava overflows from all eruptive vents in the seconds before the explosion onset, pushed out by a substantial gas pocket rising from depth. Due to magma properties, the explosion occurred at shallow depths inside the conduit and manifested as a metre-sized exploding gas bubble^56^, delaying the electrification processes. We speculate that this was further attenuated and delayed by the magmatic temperature^57^ of the erupted mass of the 3 July 2019 paroxysm, evident by the incandescence even during daylight (Fig. 1d). Additionally, the greater magnitude of the explosion would have created a well-mixed, chaotic flow with relatively small eddies, hindering the organisation of charge and the formation of clusters. On the other hand, during the major explosions fragmentation most likely occurred at a deeper level in the conduit, resulting in readily fragmented and charged material emerging from the crater.

The estimates of the charge moment magnitude from the detected discharges showed an increase with increasing plume height between the four explosions discussed in this study (Table 1). The charge clusters building up in the volcanic plume and subsequently discharged by volcanic lightning, may be proportional to the plume volume. More extensive charge regions would result in higher charge moment magnitudes and correspondingly produce larger lightning flashes.

## Conclusion

The electrical activity of three major explosions and the 3 July 2019 paroxysm at Stromboli was detected using a thunderstorm detector. Similarities in the electrostatic signal of major explosions were found: the emergence and expansion of the plume produced a movement of charge at the onset, shortly followed by a few tens of electrical discharges, with one or more discharges of higher magnitude towards the end of the explosion. We propose that these recurrent observations could supplement the classification scheme used for the explosions on Stromboli in future. In addition to these characteristics, an oscillating electrostatic signal was detected during the 19 July 2020 major explosion, which was most likely caused by shaking of the antenna due to seismicity.

The electrical measurements obtained during the 3 July 2019 paroxysm are unprecedented. The electrical activity was much more intense and complex compared to the major explosions. Four phases of electrical activity could be distinguished and were tentatively matched to different phases of the eruption. Particularly interesting is the shift in electrical activity concomitant to the partial collapse of the plume and the subsequent onset of pyroclastic density currents down the Sciara del Fuoco.

Although not further discussed in this study, normal Strombolian explosions also produced detectable electrical signals. These results show that basaltic explosions at magmatic temperatures produce detectable movements of charge and electrical discharges, which holds promises for monitoring low VEI activity at basaltic volcanoes using robust electrostatic sensors.

## Methods

### Instrumentation

To detect electrical discharges in situ, a prototype of the Biral Thunderstorm Detector BTD-200 was used. The detector consists of two grounded antennas: a primary antenna composed of a stainless steel sphere that sits at the top of the sensor and a secondary antenna consisting of a stainless steel disc plate that is situated directly below and is shielded by a black Acetal cap^58,59^ (Fig. 1c). Both antennas detect slow variations in the electrostatic field resulting from charge neutralisation due to electrical discharges^58,59^. Due to its greater surface area, the primary antenna has the highest sensitivity, while correlation and decorrelation with the secondary antenna allow for the discrimination between electrical discharges and impact transients (ash falling or charged precipitation) on the antenna^58,59^, respectively. In case of the latter, the primary antenna will produce a transient change in the electric field as charge flows to the ground upon impact, inducing a current of opposite polarity upon the shielded secondary antenna. The sensor measures within the extremely to super low frequency range (1–45 Hz) at a sample rate of 100 Hz and has been modified to allow for GPS time synchronisation and several years of data storage^59^.

The first detector, BTD1, was installed on Stromboli on 11 June 2019 and was operational for three weeks. The related data set allows for distinguishing eruptive activity of different intensities and different phases of the paroxysm. The sensor was installed northeast from the crater terrace, at a distance of 292 m from the NE and 469 m from the SW crater zone, respectively, and at an altitude of 774 m (Fig. 1a,b). Although BTD1 did not get damaged during the 3 July 2019 paroxysm (as well as the 28 August 2019 paroxysm), hot ballistics destroyed the power system, effectively cutting off power and stopping the recording 45 s after the onset of the paroxysm.

Due to the apparent volcanic hazard after the 2019 paroxysms, access was restricted to an altitude of 290 m a.s.l. A new detector, BTD2, was installed on 22 September 2019 at an altitude of ~190 m a.s.l. at the edge of the Sciara del Fuoco near Punta Labronzo, at a distance ranging between ~1.55 and 1.71 km from the craters (Fig. 1a,b).

As the intensity of the electric field decreases proportional to the distance cubed^60,61^, a closer location of the instrument to the craters was preferable but unavailable in September 2019. On 9 October 2020, BTD2 was relocated close to Roccette at a distance ranging between 425 and 616 m from each crater zone and at an altitude of 798 m, and was renamed BTD3 (Fig. 1a,b). At the time of writing, BTD3 is still up and recording continuously (Fig. 1e).

### Data processing

The analogue raw voltage output from the antennas is converted to a digital voltage (V) used for calculation by the internal processors. Note that the resulting voltage is not the actual voltage of the electrical discharge, but rather a signal proportional to the current induced by the atmospheric electric field change associated with an electrical discharge. This depends on the distance of the sensor and the height at which the discharge occurs. In general, charge movement, as a result of space charge attaching to the ejected volcanic particles and gases, will produce a relatively slow-varying electrical signal, while electrical discharges produce a transient, the steep onset and decrease indicating a short-lived, sharp change in the electric field. Electrical discharges were identified using an adjusted version of the volcanic lightning detection algorithm used at Sakurajima volcano, Japan^59^. Signals were interpreted as electrical discharges based on the following empirical thresholds:

1. The signal needed to have the same polarity at both antennas (positive covariance);
2. The positive covariance is ≥ 0.5;
3. The ratio between the two antenna signals is > 3.0;
4. The signal-to-noise ratio (SNR) is > 2.0 and > 1.1 for the primary and secondary antenna, respectively.

The covariance was calculated over a moving window of 16 samples (step size of 1 sample), while the noise was calculated over a moving window of 128 samples (step size of 1 sample). Albeit the good accuracy of the algorithm^59^, the low SNR of the secondary signal led to the omission of electrical discharges at certain times. On the other hand, attenuation of the transient caused by an electrical discharge and oscillations in the electrical signal occasionally met all criteria of the algorithm, resulting in wrongly interpreted discharges. These small inaccuracies were manually corrected.

For each explosion, the total number of discharges and the maximum discharge rate per 5 s, with a step size of 1 s, were determined. Also, the total duration of electrical activity was calculated. As it may take time for the remaining charge to dissipate after the explosion, the last discharge is used to mark the end of the electrical activity.

To compare the magnitude of the different explosions, the peak voltage of the charge movement detected by the sensor shortly after the explosion, needed to be corrected for the distance cubed between the sensor and the active crater^60,61^. This provided an estimate of the magnitude of the initial charge moved at the active crater during each explosion. Similar to the measured voltage of the discharges, this value is proportional to the current induced by the atmospheric electric field change. Given the BTD output voltage is proportional to induced current, the total charge neutralised by the discharge was therefore estimated by integrating the BTD output voltage during the discharge and using a pre-determined conversion factor based on electrostatic modelling of the sensor’s geometry and calibration during thunderstorms. This, combined with estimates of the distance to the discharge and its height above the crater, are used to estimate charge moment magnitude using the conventional vertical dipole field lightning model^61^.

These electrical measurements are compared to several eruption parameters, including acoustic pressure, maximum particle ejection velocity, plume height above the crater rim (a.c.r.) and eruption duration.

## Supplementary Information

Supplementary Information.

## Data Availability

The electrical data is available here: Vossen, C. & Cimarelli, C. Electrical measurements of explosive volcanic eruptions from Stromboli Volcano, Italy. GFZ Data Services. https://doi.org/10.5880/fidgeo.2022.005 (2022).

## References

[1] Cimarelli C, Genareau K (2021). A review of volcanic electrification of the atmosphere and volcanic lightning. J. Volcanol. Geotherm. Res..

[2] Dickinson JT (1988). Fractoemission from fused silica and sodium silicate glasses. J. Vac. Sci. Technol. A.

[3] James MR, Lane SJ, Gilbert JS (2000). Volcanic plume electrification: Experimental investigation of a fracture-charging mechanism. J. Geophys. Res. Solid Earth.

[4] Lacks DJ, Levandovsky A (2007). Effect of particle size distribution on the polarity of triboelectric charging in granular insulator systems. J. Electrost..

[5] Cimarelli C, Alatorre-Ibargüengoitia MA, Kueppers U, Scheu B, Dingwell DB (2014). Experimental generation of volcanic lightning. Geology.

[6] Gaudin D, Cimarelli C (2019). The electrification of volcanic jets and controlling parameters: A laboratory study. Earth Planet. Sci. Lett..

[7] Arason P, Bennett AJ, Burgin LE (2011). Charge mechanism of volcanic lightning revealed during the 2010 eruption of Eyjafjallajökull. J. Geophys. Res. Solid Earth.

[8] Van Eaton AR (2020). Did ice-charging generate volcanic lightning during the 2016–2017 eruption of Bogoslof volcano, Alaska?. Bull. Volcanol..

[9] Björnsson S, Blanchard DC, Spencer AT (1967). Charge generation due to contact of saline waters with molten lava. J. Geophys. Res..

[10] Büttner R, Röder H, Zimanowski B (1997). Electrical effects generated by experimental volcanic explosions. Appl. Phys. Lett..

[11] James MR (2008). Electrical charging of volcanic plumes. Space Sci. Rev..

[12] Clement CF, Harrison RG (1992). The charging of radioactive aerosols. J. Aerosol Sci..

[13] Nicoll K (2019). First in situ observations of gaseous volcanic plume electrification. Geophys. Res. Lett..

[14] McNutt SR, Williams ER (2010). Volcanic lightning: Global observations and constraints on source mechanisms. Bull. Volcanol..

[15] Houghton BF (2013). Pushing the Volcanic Explosivity Index to its limit and beyond: Constraints from exceptionally weak explosive eruptions at Kīlauea in 2008. Geology.

[16] Taddeucci J (2021). Fracturing and healing of basaltic magmas during explosive volcanic eruptions. Nat. Geosci..

[17] Colombier M (2021). Degassing and gas percolation in basaltic magmas. Earth Planet. Sci. Lett..

[18] Papale P (1999). Strain-induced magma fragmentation in explosive eruptions. Nature.

[19] Calvari S, Spampinato L, Lodato L (2006). The 5 April 2003 vulcanian paroxysmal explosion at Stromboli volcano (Italy) from field observations and thermal data. J. Volcanol. Geotherm. Res..

[20] Büttner R, Zimanowski B, Röder H (2000). Short-time electrical effects during volcanic eruption: Experiments and field measurements. J. Geophys. Res. Solid Earth.

[21] Calvari S (2012). The 7 September 2008 Vulcanian explosion at Stromboli volcano: Multiparametric characterization of the event and quantification of the ejecta. J. Geophys. Res. Solid Earth.

[22] Rosi M, Bertagnini A, Landi P (2000). Onset of the persistent activity at Stromboli volcano (Italy). Bull. Volcanol..

[23] Rosi M (2013). Stromboli volcano, Aeolian Islands (Italy): Present eruptive activity and hazards. Geol. Soc. Lond. Mem..

[24] Barberi F, Civetta L, Rosi M, Scandone R (2009). Chronology of the 2007 eruption of Stromboli and the activity of the Scientific Synthesis Group. J. Volcanol. Geotherm. Res..

[25] Schmid M (2021). Characterising vent and crater shape changes at Stromboli: Implications for risk areas. Volcanica.

[26] Houghton BF (2016). Stronger or longer: Discriminating between Hawaiian and Strombolian eruption styles. Geology.

[27] Mercalli G (1907). I Vulcani Attivi Della Terra: Morfologia, Dinamismo, Prodotti, Distribuzione Geografica, Cause.

[28] Taddeucci J (2013). Linked frequency and intensity of persistent volcanic activity at Stromboli (Italy). Geophys. Res. Lett..

[29] Francalanci, L. *et al*. Mineralogical, geochemical, and isotopic characteristics of the ejecta from the 5 April 2003 paroxysm at Stromboli, Italy: Inferences on the preeruptive magma dynamics. In *Geophys. Monogr. Ser.* American Geophysical Union. http://hdl.handle.net/2122/4635 (2008).

[30] Métrich N, Bertagnini A, Di Muro A (2010). Conditions of magma storage, degassing and ascent at Stromboli: New insights into the volcano plumbing system with inferences on the eruptive dynamics. J. Petrol..

[31] Bertagnini A (2008). Volcanology and magma geochemistry of the present-day activity: constraints on the feeding system. Learn. Stromboli Am. Geophys. Univ. Wash. Geophys. Mon..

[32] Bertagnini A, Di Roberto A, Pompilio M (2011). Paroxysmal activity at Stromboli: Lessons from the past. Bull. Volcanol..

[33] Bevilacqua A (2020). Major explosions and paroxysms at Stromboli (Italy): A new historical catalog and temporal models of occurrence with uncertainty quantification. Sci. Rep..

[34] Gurioli L (2013). Classification, landing distribution, and associated flight parameters for a bomb field emplaced during a single major explosion at Stromboli, Italy. Geology.

[35] Calvari S (2021). Variable magnitude and intensity of Strombolian explosions: Focus on the eruptive processes for a first classification scheme for Stromboli volcano (Italy). Remote Sens..

[36] Andronico D (2021). Uncovering the eruptive patterns of the 2019 double paroxysm eruption crisis of Stromboli volcano. Nat. Commun..

[37] Giordano G, De Astis G (2021). The summer 2019 basaltic Vulcanian eruptions (paroxysms) of Stromboli. Bull. Volcanol..

[38] Pistolesi M, Delle Donne D, Pioli L, Rosi M, Ripepe M (2011). The 15 March 2007 explosive crisis at Stromboli volcano, Italy: Assessing physical parameters through a multidisciplinary approach. J. Geophys. Res. Solid Earth.

[39] Gaudin D (2017). Integrating puffing and explosions in a general scheme for Strombolian-style activity. J. Geophys. Res. Solid Earth.

[40] Vossen C, Cimarelli C (2022). Electrical measurements of explosive volcanic eruptions from Stromboli Volcano, Italy. GFZ Data Serv..

[41] Istituto Nazionale di Geofisica e Vulcanologia, Sections of Catania, Napoli and Palermo. Stromboli, Bollettino Settimanale 24/06/2019–30/06/2019 [weekly bulletin, N° 27/2019]. *INGVvulcani *(2019, July 2). Retrieved from https://www.ct.ingv.it/index.php/monitoraggio-e-sorveglianza/prodotti-del-monitoraggio/bollettini-settimanali-multidisciplinari (Accessed 20 December 2021).

[42] Istituto Nazionale di Geofisica e Vulcanologia, Sections of Catania, Napoli and Palermo. Stromboli, Bollettino Settimanale 13/07/2020–19/07/2020 [weekly bulletin, N° 30/2020]. *INGVvulcani *(2020a, July 21). Retrieved from https://www.ct.ingv.it/index.php/monitoraggio-e-sorveglianza/prodotti-del-monitoraggio/bollettini-settimanali-multidisciplinari (Accessed 20 December 2021).

[43] Istituto Nazionale di Geofisica e Vulcanologia, Sections of Catania, Napoli and Palermo. Stromboli, Bollettino Settimanale 16/11/2020–22/11/2020 [weekly bulletin, N° 48/2020]. *INGVvulcani *(2020b, November 24). Retrieved from https://www.ct.ingv.it/index.php/monitoraggio-e-sorveglianza/prodotti-del-monitoraggio/bollettini-settimanali-multidisciplinari (Accessed 20 December 2021).

[44] Laboratorio Geofisica Sperimentale. Bollettino settimanale dell’attività del vulcano Stromboli 21 giugno–27 giugno 2019) [weekly bulletin] (2019a). Retrieved from http://lgs.geo.unifi.it/index.php/reports/stromboli-weekly (Accessed 15 December 2021).

[45] Laboratorio Geofisica Sperimentale. Bollettino settimanale dell’attività del vulcano Stromboli (28 giugno–4 luglio 2019) [weekly bulletin] (2019b). Retrieved from http://lgs.geo.unifi.it/index.php/reports/stromboli-weekly (Accessed 15 December 2021).

[46] Laboratorio Geofisica Sperimentale. Bollettino settimanale dell’attività del vulcano Stromboli (16 Luglio–23 Luglio 2020) [weekly bulletin] (2020a). Retrieved from http://lgs.geo.unifi.it/index.php/reports/stromboli-weekly (Accessed 15 December 2021).

[47] Laboratorio Geofisica Sperimentale. Bollettino settimanale dell’attività del vulcano Stromboli (13–19 novembre 2020) [weekly bulletin] (2020b). Retrieved from http://lgs.geo.unifi.it/index.php/reports/stromboli-weekly (Accessed 15 December 2021).

[48] Giudicepietro F (2020). Geophysical precursors of the July–August 2019 paroxysmal eruptive phase and their implications for Stromboli volcano (Italy) monitoring. Sci. Rep..

[49] Aiuppa A (2021). Volcanic CO_2_ tracks the incubation period of basaltic paroxysms. Sci. Adv..

[50] Behncke, B. The 3 July 2019 paroxysm of Stromboli and its activity during the following days [blog post]. *INGVvulcani *(2019, July 15). Retrieved from https://ingvvulcani.com/2019/07/15/the-3-july-2019-paroxysm-of-stromboli-and-its-activity-during-the-following-days/ (Accessed 15 December 2021).

[51] McNutt SR, Davis CM (2000). Lightning associated with the 1992 eruptions of Crater Peak, Mount Spurr volcano, Alaska. J. Volcanol. Geotherm. Res..

[52] Méndez Harper J, Dufek J (2016). The effects of dynamics on the triboelectrification of volcanic ash. J. Geophys. Res. Atmos..

[53] Cooray V (2015). An Introduction to Lightning.

[54] Ripepe M (2021). Ground deformation reveals the scale-invariant conduit dynamics driving explosive basaltic eruptions. Nat. Commun..

[55] MacGorman DR, Rust WD (1998). Observations of the Electrical Characteristics of Thunderstorms: Chapter 7 in the Electrical Nature of Storms.

[56] Métrich N, Bertagnini A, Pistolesi M (2021). Paroxysms at Stromboli volcano (Italy): Source, genesis and dynamics. Front. Earth Sci..

[57] Stern S, Cimarelli C, Gaudin D, Scheu B, Dingwell DB (2019). Electrification of experimental volcanic jets with varying water content and temperature. Geophys. Res. Lett..

[58] Bennett AJ (2017). Electrostatic thunderstorm detection. Weather.

[59] Vossen CEJ (2021). Long-term observation of electrical discharges during persistent Vulcanian activity. Earth Planet. Sci. Lett..

[60] Wilson CTR (1921). Investigations on lighting discharges and on the electric field of thunderstorms. Philos. Trans. R. Soc. Lond. A Contain. Pap. Math. Phys. Character.

[61] Bennett AJ, Harrison RG (2013). Lightning-induced extensive charge sheets provide long range electrostatic thunderstorm detection. Phys. Rev. Lett..

