# Supplementary material for: The electrical signature of mafic explosive eruptions at Stromboli volcano, Italy
Source: Sci Rep. 2022 May 31;12:9049. doi: 10.1038/s41598-022-12906-x (PMC9156681; doi:10.1038/s41598-022-12906-x)
Supplement: Supplementary file 1 — Supplementary Information. [file 41598_2022_12906_MOESM1_ESM.pdf]

Supplementary Material

The electrical signature of mafic explosive eruptions at Stromboli volcano, Italy

Caron E.J. Vossen[1,*], Corrado Cimarelli[1], Alec J. Bennett[2,3], Markus Schmid[1], Ulrich Kueppers[1], Tullio Ricci[4], Jacopo Taddeucci[4]

[1] Department of Earth and Environmental Sciences, Ludwig-Maximilians-Universität München, Theresienstraße 41, 80333 Munich, Germany
[2] Bristol Industrial and Research Associates Ltd (Biral), Unit 8 Harbour Road Trading Estate, Portishead, Bristol, BS20 7BL, United Kingdom
[3] Department of Electronic and Electrical Engineering, University of Bath, Claverton Down, Bath, BA2 7AY, United Kingdom
[4] Istituto Nazionale di Geofisica e Vulcanologia, Sezione di Roma 1, Roma, Italy

[*] Corresponding author. E-mail address: caron.vossen@min.uni-muenchen.de

Supplementary Figure S1 shows the electrical signals recorded by BTD1 during three types of Strombolian explosions according to the classification scheme proposed by Gaudin et al. (2017): Type 1 is bomb-dominated; Type 2a is dominated by both ash and bombs; Type 2b is ash-dominated (Patrick et al., 2007).

For each explosion, there was no variation detected in the secondary signal, indicating that the secondary antenna is not sensitive enough to detect any electrical activity during the Strombolian explosions. On the other hand, the primary antenna does show electric field gradients, visible as slow (~1-3 s) variations in the electrical signal. Additionally, several transients were detected during Type 2a and 2b explosions, which can be interpreted as small electrical discharges.

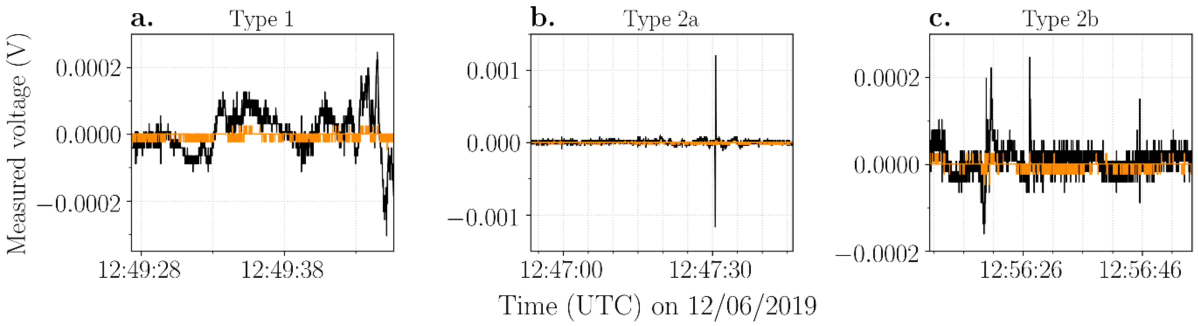

Supplementary Figure S1: Voltages (V) measured by the primary (black line) and secondary (orange line) antennas of BTD1 on 12 June 2019. ***a)*** *Strombolian explosion of Type 1.* ***b)*** *Strombolian explosion of Type 2a.* ***c)*** *Strombolian explosion of Type 2b. The time axes correspond to the duration of the explosions, during which pyroclasts were actively ejected.*

Supplemental References

Gaudin, D. *et al*. Integrating puffing and explosions in a general scheme for Strombolian-style activity. *J. Geophys. Res. Solid Earth*, **122**, 1860-1875; https://doi.org/10.1002/2016JB013707 (2017).

Patrick, M. R. *et al*. Strombolian explosive styles and source conditions: insights from thermal (FLIR) video. *Bull. Volcanol.*, **69**, 769-784; https://doi.org/10.1007/s00445-006-0107-0 (2007).
